# Myopia: an emerging public health challenge in South Asia

**Published:** 2019-05-13

**Authors:** Srinivas Marmamula, Navya Rekha Barrenkala

**Affiliations:** 1 Associate Director: Public Health - Research and Training, Gullapalli Pratibha Rao International Centre for Advancement of Rural Eye Care, L V Prasad Eye Institute, India.; 2 Research Optometrist: Gullapalli Pratibha Rao International Centre for Advancement of Rural Eye care, L V Prasad Eye Institute, Hyderabad, India.

**Uncorrected refractive errors (URE), predominantly myopia, remains the leading cause of vision loss worldwide including in South Asia.**

**Figure:**
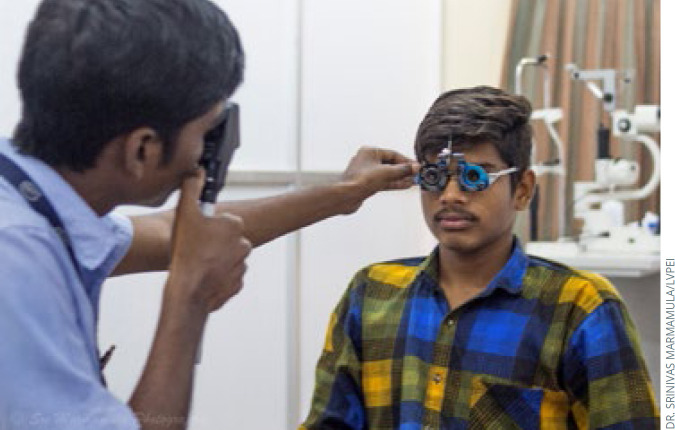
Refraction being performed at a vision centre. INDIA

Vision impairment (VI) is a global public health challenge that affects an estimated 253 million people.^1^ Uncorrected refractive errors (URE), predominantly myopia remains the leading cause of vision loss worldwide including South Asia (SA).^2^ Myopia or near-sightedness, is a refractive error, a condition in which the eye does not bend or refract light properly. This means that while close objects look clear, distant objects look blurred. Although the definition of myopia in terms of magnitude varies across the studies, it is measured in dioptres.

According to projections reported by Holden et al it is estimated that URE and mainly myopia was the most common cause of distance vision impairment affecting over 108 million people worldwide in the year 2010.^3^ Recent meta-analysis reported a prevalence of myopia (spherical equivalent of 0.5 D or less myopia) as 20.2 per cent in 2010 and is projected to increase to 28.6 per cent by 2020 and 53 per cent by 2050.^3^ It is also estimated that by 2050, 50 per cent of the global population will be myopic and 10 per cent will have high myopia.^3^ It is evident that myopia is an emerging public health challenge that needs to be addressed worldwide.

The South Asia region comprises eight countries, including India, Pakistan and Bangladesh, three of the 10 most populated countries in the world. The region hosts 23 per cent of the world population and shares a disproportionately large burden of 30 per cent of the global VI.^1^ While URE contributes to 50 per cent of the global VI, in this region it is as high as 63 per cent.^2^

## Myopia in children and young adults

The Refractive Errors Studies in Children (RESC) reported a higher prevalence of myopia in children from urban India (Delhi) compared to rural India (Mahbubnagar). The prevalence of refractive errors in rural Nepal was the least (1.2 per cent). This two-to three-fold higher prevalence of myopia in urban children is predominantly due to increased near work activities. A recent longitudinal study conducted among children in India revealed that an increase in near-work activities such as reading and writing per week, excessive use of electronic gadgets such as computers, tablets, video games and watching television were significant risk factors for progression of myopia.^4^ The same study also revealed that outdoor activities or time spent outdoors (>2 hours) were protective against progression of myopia. There is also evidence from studies done in other regions of the world showing that an increase in outdoor activity may have a protective effect on onset and progression of myopia. An increase in outdoor activities for children and a restriction on screen-time can be a useful public health intervention for myopia in this region.

## Myopia in older populations

The prevalence of myopia in older adults is highly variable in the region ranging from close to 20 per cent in central India to 43 per cent in Myanmar. This large variability is due to several factors including the age of the participants and other risk factors such as ethnicity. Some myopia in the older age groups may be due to nuclear cataract (cataract in the central zone (nucleus) of the lens). A good cataract surgery programme will be able to address myopia caused due to cataract.

## Models for correcting myopia

Addressing the myopia challenge in SA needs a multi-pronged approach based on early detection and appropriate correction. As school children form a captive group, school-based eye health programmes or camps can detect myopia in children and provide appropriate correction. Community-based screening programmes (CSP) are often conducted by non-government organisations in this region. The CSPs are conducted as “make-shift clinics” set-up in areas where limited eye care services are available. All those visiting these clinics are screened for vision impairment. Those with refractive errors are provided spectacles and those with uncorrectable vision loss are referred to a base hospital where services including cataract surgery are provided. The vision centre (VC) model of primary eye care service delivery has evolved in India and now spread to other countries with relevant local modifications.

**Figure:**
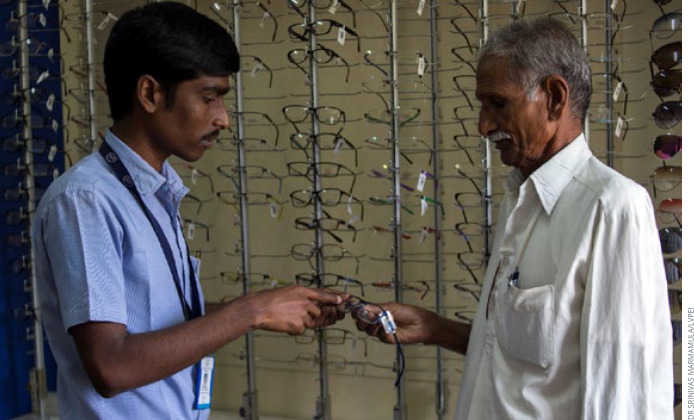
Dispensing spectacles at a vision centre. INDIA

## Modalities for correcting myopia in SA

Myopia is considered to be a correctable form of vision loss. Spectacles remain the mainstay for correction of myopia in the SA region. However, with new technological innovations and more predictable outcomes, refractive surgery is increasing especially in urban areas. Recognising the importance of preventing the progression of myopia, several modes of prevention are being tried. Orthokeratology (ortho-k) reverse geometry contact lenses are one such modality. These contact lenses worn overnight temporarily aim to reshape the corneal surface. Pharmacological interventions to reduce myopia and axial elongation have been evaluated in a few studies.

This issue of the South Asia edition will provide a comprehensive review of myopia in the region. Ravilla et al provide an overview on spectacle dispensing at the community level. Acharya et al describe the current trends in surgical options for correction of myopia. Murthy et al describe the models of myopia correction at a population level. Verkicherla et al highlight new promising technologies and artificial intelligence in early detection and management of myopia in the region. These models for correction of myopia and application of innovation and technology to address the myopia challenge are also presented.

## Acknowledgements

The authors wish to thank Shashank Yellapragada and Dr Rohit C Khanna for contributing to this article.
